# Guidelines on the risk for transmission of infectious agents during xenotransplants.

**DOI:** 10.3201/eid0104.950412

**Published:** 1995

**Authors:** L E Chapman


### Guidelines on the Risk for Transmission of Infectious Agents During Xenotransplants

An increasingly critical shortage of human donors has limited the availability and benefit of organ and tissue transplantation. This chronic shortage, coupled with recent scientific and biotechnological advances, has been a catalyst for new therapeutic approaches directed at using animal tissues in humans. The use of xenogeneic tissues and organs for transplantation or perfusion has raised concerns about the potential of both recognized zoonotic pathogens and unknown xenogeneic agents to infect individual human recipients and then spread through human populations.

Public health guidelines intended to minimize the risk for transmission of known pathogens through human-to-human transplantation exist. Similar guidelines addressing the issue of infectious agents that may be associated with xenotransplantation are being jointly developed by Public Health Service working groups at the Centers for Disease Control and Prevention (CDC), the Food and Drug Administration, and the National Institutes of Health. A provisional draft of these guidelines will be published in the Federal Register in late 1995. Public comment on the proposed guidelines is invited. Critical review by members of the transplant community is particularly sought. Publication of a final version of these guidelines in CDC's *Morbidity and Mortality Weekly Report* is planned for the spring of 1996.

**Louisa E. Chapman**
National Center for Infectious Diseases
Centers for Disease Control and Prevention
Atlanta, Georgia, USA

### Emerging Infectious Diseases Featured at ICAAC/IDSA Meeting

Emerging infectious diseases were highlighted recently at a joint meeting of the Interscience Conference on Antimicrobial Agents and Chemotherapy (ICAAC) and the Infectious Diseases Society of America (IDSA) in San Francisco. In his opening address, IDSA President Vincent Andriole stated that the topic of most pressing concern to both organizations was new and reemerging pathogens. Presentations were made on the following subjects: arenavirus hemorrhagic fevers; changing virulence of streptococcal infections; cryptosporidia, cyclospora, and microsporidia; dengue/dengue hemorrhagic fever; emerging fungal pathogens; epidemic diphtheria in the newly independent states; *Escherichia coli* O157:H7; *Helicobacter pylori*; human ehrlichioses; new lymphotropic herpesviruses; and rabies.

Common themes emanated from the presentations. The speakers noted that infectious diseases continue to occur throughout the world, both sporadically and as outbreaks, because of multiple factors. They observed that the incidence and prevalence of infectious diseases are increasing in certain populations, particularly among immunocompromised persons. Additionally, new infectious diseases and etiologic agents continue to be identified with remarkable frequency, and microorganisms are being identified as causes of chronic diseases, including cancer. Several presenters expressed concern about the migrations of animal reservoirs and arthropod vectors into new populations and geographic areas. The speakers also called for additional support for the public health infrastructure and for basic sciences that provide the foundation for infectious disease prevention and treatment.

### Building a Geographic Information System (GIS) Public Health Infrastructure for Research and Control of Tropical Diseases

A course on using Atlas GIS software and associated peripherals, such as digitizing tablets and global positioning systems (GPS), to build a GIS public health infrastructure in Latin American countries was taught August 7 to 18, 1995, at the Centers for Disease Control field station in Guatemala City, which includes the Medical Entomology Research Training Unit housed at the Universidad del Valle de Guatemala. The course was funded by the Special Programme on Research and Training in Tropical Diseases and presented by the Latin American Tropical Disease Research Training Consortium. Course

